# SOD1 inhibition enhances sorafenib efficacy in HBV‐related hepatocellular carcinoma by modulating PI3K/Akt/mTOR pathway and ROS‐mediated cell death

**DOI:** 10.1111/jcmm.18533

**Published:** 2024-07-21

**Authors:** Jooyoung Lee, Jiye Kim, Ryunjin Lee, Eunkyeong Lee, Hye‐In An, Yong‐Jae Kwon, Hana Jin, Chan‐Gi Pack, Inki Kim, Young‐In Yoon, Gil‐Chun Park, Eun‐Kyoung Jwa, Jae Hyun Kwon, Jung‐Man Namgoong, Gi‐Won Song, Shin Hwang, Eunyoung Tak, Sung‐Gyu Lee

**Affiliations:** ^1^ Asan Institute for Life Sciences, Asan Medical Center University of Ulsan College of Medicine Seoul South Korea; ^2^ Department of Biochemistry and Molecular Biology, AMIST, Asan Medical Center University of Ulsan College of Medicine Seoul South Korea; ^3^ Department of Surgery, Gangneung Asan Hospital University of Ulsan College of Medicine Seoul South Korea; ^4^ Division of Vascular Surgery, Department of Surgery, Asan Medical Center University of Ulsan College of Medicine Seoul South Korea; ^5^ Convergence Medicine Research Center (CREDIT) Asan Institute for Life Sciences, ASAN Medical Center Seoul Republic of Korea; ^6^ Division of Hepatobiliary Surgery and Liver Transplantation, Department of Surgery, Asan Medical Center University of Ulsan College of Medicine Seoul South Korea; ^7^ Department of Surgery, Hallym University Sacred Heart Hospital Hallym University College of Medicine Anyang South Korea; ^8^ Division of Pediatric Surgery, Department of Surgery, Asan Medical Center University of Ulsan College of Medicine Seoul South Korea

**Keywords:** disulfiram, hepatitis B virus, hepatocellular carcinoma, Sorafenib resistance, superoxide dismutase

## Abstract

Hepatitis B Virus (HBV) infection significantly elevates the risk of hepatocellular carcinoma (HCC), with the HBV X protein (HBx) playing a crucial role in cancer progression. Sorafenib, the primary therapy for advanced HCC, shows limited effectiveness in HBV‐infected patients due to HBx‐related resistance. Numerous studies have explored combination therapies to overcome this resistance. Sodium diethyldithiocarbamate (DDC), known for its anticancer effects and its inhibition of superoxide dismutase 1 (SOD1), is hypothesized to counteract sorafenib (SF) resistance in HBV‐positive HCCs. Our research demonstrates that combining DDC with SF significantly reduces HBx and SOD1 expressions in HBV‐positive HCC cells and human tissues. This combination therapy disrupts the PI3K/Akt/mTOR signalling pathway and promotes apoptosis by increasing reactive oxygen species (ROS) levels. These cellular changes lead to reduced tumour viability and enhanced sensitivity to SF, as evidenced by the synergistic suppression of tumour growth in xenograft models. Additionally, DDC‐mediated suppression of SOD1 further enhances SF sensitivity in HBV‐positive HCC cells and xenografted animals, thereby inhibiting cancer progression more effectively. These findings suggest that the DDC‐SF combination could serve as a promising strategy for overcoming SF resistance in HBV‐related HCC, potentially optimizing therapy outcomes.

## 1 INTRODUCTION

Hepatocellular carcinoma (HCC) is a major global health concern and is the sixth leading cause of cancer‐related death worldwide.^1^ Among the population of patients with chronic hepatitis B virus (HBV), HCC poses an even greater challenge,^2^ with an estimated 257 million individuals infected with HBV globally in 2018.^3^ HBV belongs to the Hepadnaviridae family and causes acute and chronic hepatitis by infecting the human liver. Despite the advancements in vaccines and therapeutic agents, current treatment strategies for HBV‐infected patients primarily focus on reducing viral activity rather than achieving complete viral elimination.^4^ The difficulty arises from the persistence of integrated HBV DNA and transcriptionally inactive cccDNA.^5^ Notably, a significant portion of the Korean population^6^ and approximately 5%–8% of the Chinese population^7^ are affected by chronic HBV infections, which can potentially lead to the development of HCC, hepatitis and cirrhosis, resulting in increased mortality rates.^8^

HBV‐related HCC is influenced by a complex interplay of viral and host factors.^9^ Upon HBV infection, certain viral proteins such as HBx contribute to oncogenic processes characterized by dysregulated cell proliferation and evasive apoptosis mechanisms. Notably, the activation of the PI3K/Akt/mTOR pathway plays a significant role in promoting cell survival and growth in HBV‐infected cells.^10^, ^11^ Chronic HBV infection leads to sustained inflammation, liver injury, and fibrosis, culminating in cirrhosis, a major risk factor for HCC. Gaining a comprehensive understanding of these mechanisms, particularly the intricate involvement of the PI3K/Akt/mTOR pathway, is crucial for the development of effective prevention and treatment strategies targeting HBV‐related HCC.

Sorafenib (SF) (brand name Nexavar™) is a standard therapy for HCC, including HCC. It acts by targeting multiple signalling pathways in tumour cells and blood vessels.^12^ However, the development of SF resistance in HBV‐related liver tumours presents a major hurdle in achieving successful outcomes through standard therapy.^13^ Therefore, finding effective treatment strategies for this subset of patients has become a critical area of research and clinical focus.

Recent studies have highlighted the potential of disulfiram (brand name Antabuse™), an FDA‐approved drug primarily used to treat alcohol addiction, in suppressing tumour growth and inhibiting viral replication.^14^, ^15^ Disulfiram has shown effectiveness against HCC and has the potential to target cancer stem cells, presenting a novel approach to preventing tumour recurrence and metastasis.^16^ Notably, disulfiram derivatives, such as DDC, have been found to inhibit Superoxide Dismutase 1 (SOD1), a protein associated with cancer cell survival and a current drawback in cancer treatment strategies.

Given the promising results of DDC in inhibiting SOD1 and its potential implications for cancer therapy, including HBV‐related HCC, this study explores the combination of sorafenib and DDC as a therapeutic regimen. The present study focuses on the combination of SF and a disulfiram derivative, DDC, as a therapeutic regimen for the treatment of HBV‐related HCC. The findings from this research have the potential to provide valuable insights into the treatment of this complex disease, leading to improved long‐term survival rates.

## 2 METHODS

### 2.1 Patient tissue samples

Thirteen individual human HCC specimens were collected from patients who underwent hepatobiliary surgery at the Division of Liver Transplantation and Hepatobiliary Surgery in Asan Medical Center (Seoul, South Korea). Small fragments of the tumour were promptly frozen in liquid nitrogen and stored at −80°C until they were used for experimentation. The Institutional Review Board (IRB) of Asan Medical Center reviewed and granted approval for the collection and utilization of patient specimens (Approval no. 2020–1464). All research was conducted in accordance with both the Declarations of Helsinki and Istanbul. All patients who provided tissue samples willingly donated their specimens and provided written informed consent. Clinical information of the participants is listed in Table S1.

### 2.2 Cell culture

The HepG2.2.15 cell line was generously provided by Dr. Eui‐Cheol Shin's lab at Korea Advanced Institute of Science and Technology (KAIST, Daejeon, South Korea).^17^ HBV‐negative HCC cell lines, including hepatoblastoma‐derived HepG2 (ATCC No. HB‐8065),^18^ liver sinusoidal endothelial cell‐derived SK‐HEP1 (ATCC No. HTB‐52),^19^ and adult HCC Huh‐7 (KCLB No. 60104), as well as the HBV‐related HCC cell lines, paediatric HCC Hep3B (ATCC No. HB‐8064) and adult HCC SNU‐449 (KCLB No. 00449), were obtained from the Korean Cell Line Bank (KCLB; Korean Cell Line Research Foundation, Seoul, South Korea) or the American Type Culture Collection (ATCC; Manassas, VA, USA). All cell lines were cultured in DMEM with high glucose (Cat. No. SH30022.01; HyClone, Cytiva, Marlborough, MA, USA) for HepG2, HepG2.2.15, SK‐HEP1, and Hep3B, or RPMI 1640 (Cat. No. SH30027.01; HyClone) for Huh‐7 and SNU‐449. The culture media were supplemented with 10% fetal bovine serum (FBS; Cat. No. F0600‐050; GenDEPOT, Barker, TX, USA), 1% streptomycin/penicillin (Cat. No. SV30010.01; HyClone), and 0.2% Normocin (Cat. No. ANT‐NR‐2; InvivoGen, San Diego, CA, USA). The cells were maintained in an incubator at 37°C in a 5% CO_2_ humidified environment.

### 2.3 Cell viability analysis

HepG2.2.15 cells were seeded in a 96‐well, flat‐bottomed microplate (Cat. No. 167008; Nunc, Thermo Fisher Scientific, Waltham, MA, USA) at a volume of 100 μL per well (0.8 × 10^5^ cells/ml) and incubated overnight in a growth medium to facilitate cell adhesion. On the following day, the growth medium was replaced with fresh media, and the cells were treated with various concentrations of DDC alone and in combination with SF. The treated cells were then incubated for up to 24 h in a 5% CO_2_ humidified environment at 37°C. After the incubation period, 10 μL of Cell Counting Kit‐8 (CCK‐8; CK04‐13, Dojindo Laboratories, Kumamoto, Japan) solution was added to each well. Following a four‐hour incubation in a 5% CO_2_ humidified environment at 37°C, the cytotoxicity of the drugs was determined by measuring the absorbance at 450 nm using a Sunrise™ spectrophotometer (Tecan, Männedorf, Switzerland).

### 2.4 Immunoblotting assay

Protein expression was assessed using the Western blot technique. HepG2.2.15 cells were treated with DDC, SF, or their combination for up to 24 h. To obtain the cell lysate, the cells were washed twice with DPBS and then extracted using RIPA buffer (50 mM Tris–HCl, pH 8.0, 1% NP‐40, 0.5% sodium deoxycholate, 150 mM NaCl, and 0.1% sodium dodecyl sulfate) supplemented with a protease and phosphatase inhibitor cocktail (Cat. No. PPC1010; Sigma‐Aldrich, Merck, Darmstadt, Germany). Human tissue samples were lysed in T‐PER buffer (Cat. No. 78510; Thermo Fisher Scientific) containing protease and phosphatase inhibitors. Protein extraction was carried out by centrifugation at 16,000 × g for 15 min at 4°C. Protein concentrations were determined using the BCA Protein Assay Reagent (Cat. No. 23225; Thermo Fisher Scientific). Equivalent amounts of protein from each sample were loaded onto polyacrylamide gels and separated through electrophoresis.

The separated proteins were then transferred to nitrocellulose membranes (Cat. No. 1704270; Bio‐Rad, Hercules, CA, USA). Blotting was performed using the TransBlot Turbo system (Bio‐Rad) for 20 min. Subsequently, the membranes were blocked with 5% skim milk dissolved in Tris‐buffered saline containing 0.1% Tween‐20 (TBST) for 1 h at room temperature. After washing, the membranes were incubated overnight at 4°C with specific primary antibodies of interest, appropriately diluted with 5% BSA in TBST. Following primary antibody incubation, the membranes were probed with horseradish peroxidase (HRP)‐conjugated anti‐mouse IgG or anti‐rabbit IgG antibodies for 1 h at room temperature (Table S2).

Protein bands were developed using ECL SuperSignal™ West Femto Maximum Sensitivity Substrate (Cat. No. 34095; Thermo Fisher Scientific, Waltham, MA, USA), and images were captured using the LuminoGraph II system (Cat. No. WSE‐6200; ATTO, Tokyo, Japan).

### 2.5 RNA interference

Control and SOD1 siRNA were obtained from Bioneer (Daejeon, South Korea). HepG2.2.15 cells were seeded onto a 6‐well plate and treated with siRNA using the Lipofectamine 2000 transfection system (Cat. No. 11668–500; Invitrogen™, Thermo Fisher Scientific) following the product guidelines. To assess the efficacy of siRNA‐mediated knockdown, we conducted a quantitative reverse transcription polymerase chain reaction (qRT‐PCR) to quantify the mRNA levels of SOD1.

### 2.6 Total RNA extraction and qRT‐PCR

Total RNA was extracted from the cells using QIAZOL reagent (Cat. No. 79306; Qiagen, Germany), followed by phase separation using chloroform. The RNA samples were then purified using a silica column‐based method (RNeasy Plus Mini Kit; Cat. No. 74136; Qiagen). The concentration and purity of the extracted RNA were determined using the Nanodrop 2000 spectrophotometer. Subsequently, cDNA synthesis was performed using the ReverTra RT master mix (Cat. No. FSQ‐301; Toyobo, Japan), facilitating the reverse transcription of RNA into complementary DNA (cDNA). The qRT‐PCR analysis was conducted using the FIREPol EvaGreen qPCR Supermix (Cat. No. 08–36‐00001; Solis BioDyne, Tartu, Estonia) and the fluorescence intensity was quantified using the CFX Connect Real‐Time PCR system (Cat. No. 1855201; Bio‐Rad). To normalize the gene expression levels, the housekeeping gene *GAPDH* was employed as an internal control using the 2^−ΔΔCt^ method.

### 2.7 Reactive oxygen species measurement

To assess intracellular reactive oxygen species (ROS) generation in HepG2.2.15 cells following treatment, we used the H_2_DCFDA cellular ROS assay kit from Abcam (Cat. No. ab113851; Abcam), following the manufacturer's instructions. The cells were treated with DDC, SF or their combination for 24 h in a 5% CO_2_ humidified environment at 37°C. After the treatment period, 20 μM of H_2_DCFDA in pre‐incubated DPBS was added to the cells and incubated for 30 min The relative intensities of green fluorescence in the different treatment groups were captured using the EVOS imaging system (Thermo Fisher Scientific). Fluorescence intensities were measured using ImageJ software (NIH, USA), and a histogram was prepared to compare the relative fluorescence intensities among the treatment groups.

### 2.8 Morphological assessment of apoptosis

To assess morphological changes indicative of apoptosis in HepG2.2.15 cells, we employed a microscopy‐based approach utilizing the Annexin V‐FITC kit (Cat. No. ab14085; Abcam). HepG2.2.15 cells were initially seeded in 6‐well plates and incubated for 24 h in a 5% CO_2_ humidified environment at 37°C to facilitate cell adherence and growth. Following the incubation period, the cells were treated with DDC, SF, or a combination of both for 24 h. Subsequently, the treated cells were stained with Annexin V‐FITC and Propidium Iodide (PI) to visualize apoptotic and dead cells. In addition to Annexin V, the cellular nucleus was stained using NucBlue™ Live Cell Stain (Cat. No. R37605; Thermo Fisher Scientific) following the manufacturer's instructions. The stained cells were then visualized using the EVOS system (Thermo Fisher Scientific).

### 2.9 Animal models

The NOD‐*Rag2* ^−/‐^ *Il2rg* ^−/−^ (NRG) immune‐deficient male mice (aged 7–8 weeks) were obtained from JA BIO (Gyeonggi‐do, South Korea) for in vivo experiments. All animal procedures were approved by the Animal Research Committee of Asan Medical Institute for Life Sciences at Asan Medical Center (Seoul, South Korea) in accordance with the guidelines outlined in the Guide for Care and Use of Laboratory Animals (IACUC Approval no. 2019–13‐071).

To evaluate the antitumor effect of DDC in combination with SF, we injected HepG2.2.15 cells (1 × 10^6^ cells/0.2 mL) into the liver parenchyma to establish liver orthotopic tumour xenografts. Mice were randomly divided into four groups (*n* = 8/group) and subjected to the following treatments: (a) vehicle control, (b) DDC (50 mg/kg/day, orally), (c) SF (SF; 40 mg/kg/day, orally), or (d) DDC plus SF (SF + DDC), administered for a duration of 3 weeks. At the end of the treatment period, the mice were euthanized in a humane manner, and liver tissues were excised and processed for tumour regression analysis.

To monitor liver function, whole‐blood samples were collected from the inferior vena cava, allowed to coagulate in serum separator tubes (SSTs; Becton Dickinson and Company), and processed. After incubation at room temperature (22 ± 2°C) for 30 min, the SST tubes were centrifuged at 2500 × g for 20 min at 4°C. The supernatants were collected as serum samples and analysed for serum alanine aminotransferase (ALT) and aspartate aminotransferase (AST) levels using a Hitachi 7180 autoanalyser (Tokyo, Japan).

### 2.10 Immunohistochemistry

Liver sections of xenografted mice were prepared for IHC staining as follows: Sections were first deparaffinized in xylene and rehydrated through an alcohol gradient. Antigen retrieval was achieved by boiling in 10 mM citrate buffer (pH 6.0) for 20 min. Endogenous peroxidase activity was quenched with 0.3% hydrogen peroxide in methanol. Sections were then treated with primary antibodies against Bcl‐2, Bax and Ki‐67, diluted in Dako REAL Antibody Diluent (Cat no. S202230‐2; Agilent Technologies). Incubation occurred overnight at 4°C. HRP signals were visualized using the DAB^+^ substrate kit (Cat no. K346811‐2; Agilent Technologies). Finally, sections were counterstained with haematoxylin QS (Vector Laboratories), mounted with VectaMount (Vector Laboratories), and imaged with an Olympus DP27 camera on an Olympus microscope at 100× magnification. Areas of positive staining were quantified using QuPath software, an open‐source platform designed for digital pathology and image analysis.^20^

### 2.11 Statistical analysis

The data presented in this study are presented as the mean ± standard deviation (SD). Statistical analysis was performed using one‐way analysis of variance (ANOVA) followed by Bonferroni's post hoc test to assess differences between the individual treatment groups and the combination treatment group. The Student's *t*‐test was employed for two‐group comparisons. A significance level of *p* < 0.05 was considered statistically significant. All analyses were performed using GraphPad Prism 8 software (GraphPad, La Jolla, CA).

## 3 RESULTS

### 3.1 HBV‐infected HCC cell lines and human specimens exhibit elevated SOD1 expression

**FIGURE 1:**
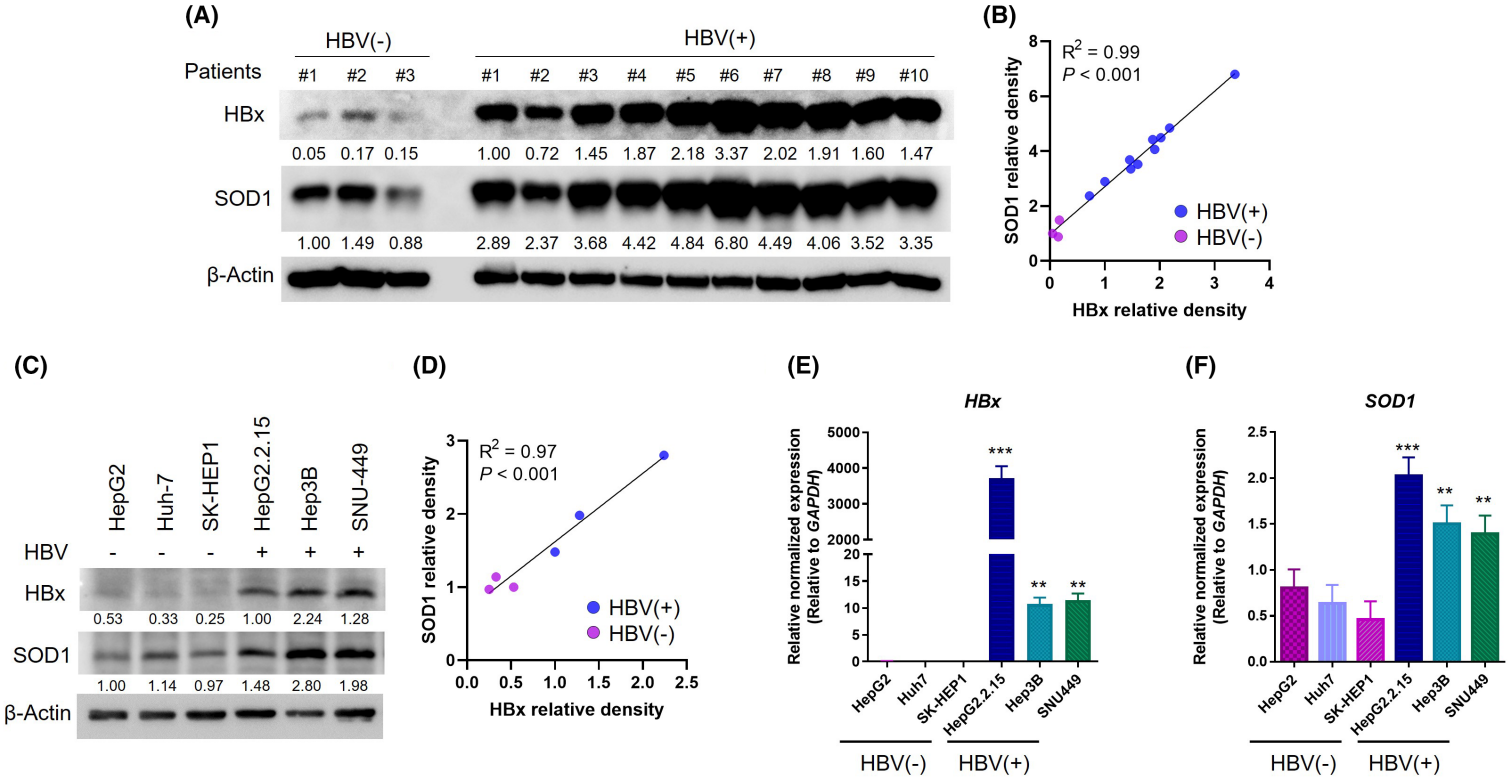
Basal expression of HBx and SOD1 in HCC cell lines and human tissue samples. (A) Immunoblots and relative quantitative analysis of human tissue proteins from HCC patients, categorized into HBV‐negative and HBV‐positive groups. The levels of HBx and SOD1 proteins were analysed. (B) Correlation between HBx and SOD1 protein expression in human HCC tissues (*p* < 0.001). (C) Basal expression levels of HBx and SOD1 proteins in HBV‐negative (HepG2, Huh‐7, SK‐Hep1) and HBV‐positive (HepG2.2.15, Hep3B, SNU‐449) HCC cells. (D) Correlation between the expression of HBx and SOD1 proteins (*p* < 0.001). (E, F) Relative gene expression of *HBx* (E) and *SOD1* (F) in HBV‐negative and HBV‐positive cell lines Significance was determined using one‐way ANOVA with Bonferroni's multiple comparisons test, with *p* < 0.05 considered significant. *p < 0.05; **p < 0.01; and ***p < 0.001.

In this study, we investigated the expression of SOD1 in both HBV‐infected patient's liver tissues and HCC cell lines. Our examination of HCC patients with HBV infection demonstrated a correlation between elevated HBx expression in liver tumour samples and tumoral SOD1 expression (Figure 1A). In liver tumour samples from non‐HBV patients, the expression of HBx was significantly lower (0.12 ± 0.04) compared to HBV‐positive tumours (1.76 ± 0.23) (*p =* 0.003). Similarly, the expression of SOD1 in non‐HBV tumours (1.12 ± 0.19) was significantly lower than in HBV‐positive tumours (4.04 ± 0.39) (*p =* 0.002). A correlation pattern similar to that observed in cell lines was also observed in patient tumour samples, with an even stronger correlation between HBx and SOD1 expression (*R* ^2^ = 0.99, *p* < 0.001; Figure 1B).

The HCC cell lines HepG2.2.15, Hep3B, and SNU‐449, which harbour integrated HBV genes, are known to express high levels of HBx protein, a key player in HBV infection (Figure 1C). Additionally, we consistently observed increased expression of SOD1, a crucial antioxidant enzyme, at both the transcript and protein levels in HBV‐infected HCC cells (Figure 1C). In HBV‐positive HCC cells, the relative density of HBx protein (1.51 ± 0.38) was significantly higher than in HBV‐negative cells (0.37 ± 0.08) (*p* = 0.042). SOD1 protein levels were also assessed, revealing higher expression in HBV‐positive cells, although the difference between HBV‐positive (2.09 ± 0.38) and HBV‐negative (1.04 ± 0.05) cells did not reach the prespecified threshold for statistical significance (*p =* 0.054). Intriguingly, a strong positive correlation was observed between HBx and SOD1 expression (*R* ^2^ = 0.97, *p* < 0.001; Figure 1D). Our analysis also revealed that these HBV‐infected cell lines exhibited significantly higher transcript levels of HBx and SOD1, compared to non‐HBV‐expressing HCC‐associated cell lines (Figure 1E, F). Considering that HBV‐positive HCCs have previously been reported to exhibit resistance to SF,^13^ the elevated levels of SOD1 observed in this study may provide a valuable clue in understanding the mechanisms underlying this resistance.

### 3.2 SF response and PI3K/Akt/mTOR pathway activation in HBV‐infected HCC cells

**FIGURE 2:**
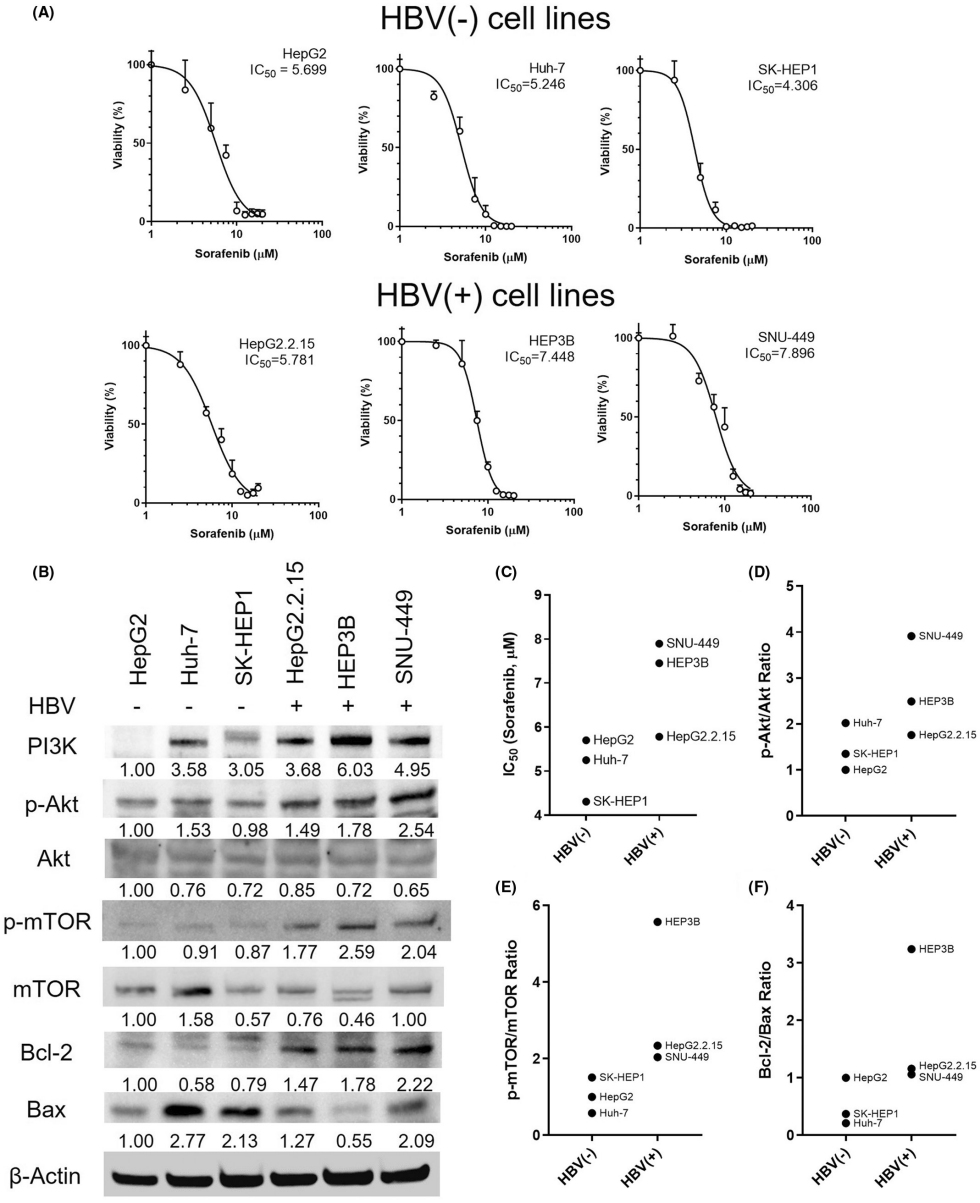
Sorafenib sensitivity and PI3K/Akt/mTOR activation in HCC cell lines. (A) WST‐8 cell viability assay and IC_50_ calculations in HBV‐negative (HepG2, Huh‐7, SK‐Hep1) and HBV‐positive (HepG2.2.15, Hep3B, SNU‐449) HCC cell lines after 24‐hour treatment with varying concentrations of sorafenib (0–20 μM). (B) Immunoblots and relative quantitative analysis of PI3K/Akt/mTOR pathway activation and the Bcl‐2/Bax ratio in HBV‐negative and HBV‐positive HCC cell lines, normalized to β‐Actin. (C) Average IC_50_ values for HBV‐negative and HBV‐positive cells. (D) Average p‐Akt/Akt ratio, (E) p‐mTOR/mTOR ratio, and (F) Bcl‐2/Bax ratio. Significance was determined using Student's *t*‐test, with *p* < 0.05 considered significant. *p < 0.05; **p < 0.01; and ***p < 0.001.

The effectiveness of SF in treating HCC is dependent on the activation of the PI3K/Akt/mTOR pathway, which is a well‐established contributor to drug resistance. Given that the HBx protein, which is commonly found in HBV‐infected liver cells, can interact with the PI3K pathway, we investigated the interplay between SF response and PI3K pathway activation in both HBV‐negative and HBV‐positive cells. To evaluate the impact of HBV integration and HBx expression on cell death in response to SF treatment, we measured the IC_50_ values for HBV‐negative and HBV‐positive HCC cell lines (Figure 2A). Notably, the IC_50_ value for HepG2.2.15 (IC_50_ = 5.781) did not show a significant increase in SF resistance when compared to HBx‐negative HepG2 cells (IC_50_ = 5.699). In contrast, HBx‐positive Hep3B (IC_50_ = 7.448) and SNU‐449 (IC_50_ = 7.896) cells exhibited higher IC_50_ values, indicating greater resistance to SF compared to HBV‐negative SK‐Hep1 (IC_50_ = 4.306) and Huh‐7 (IC_50_ = 5.246) cells (Figure 2C).

**FIGURE 3:**
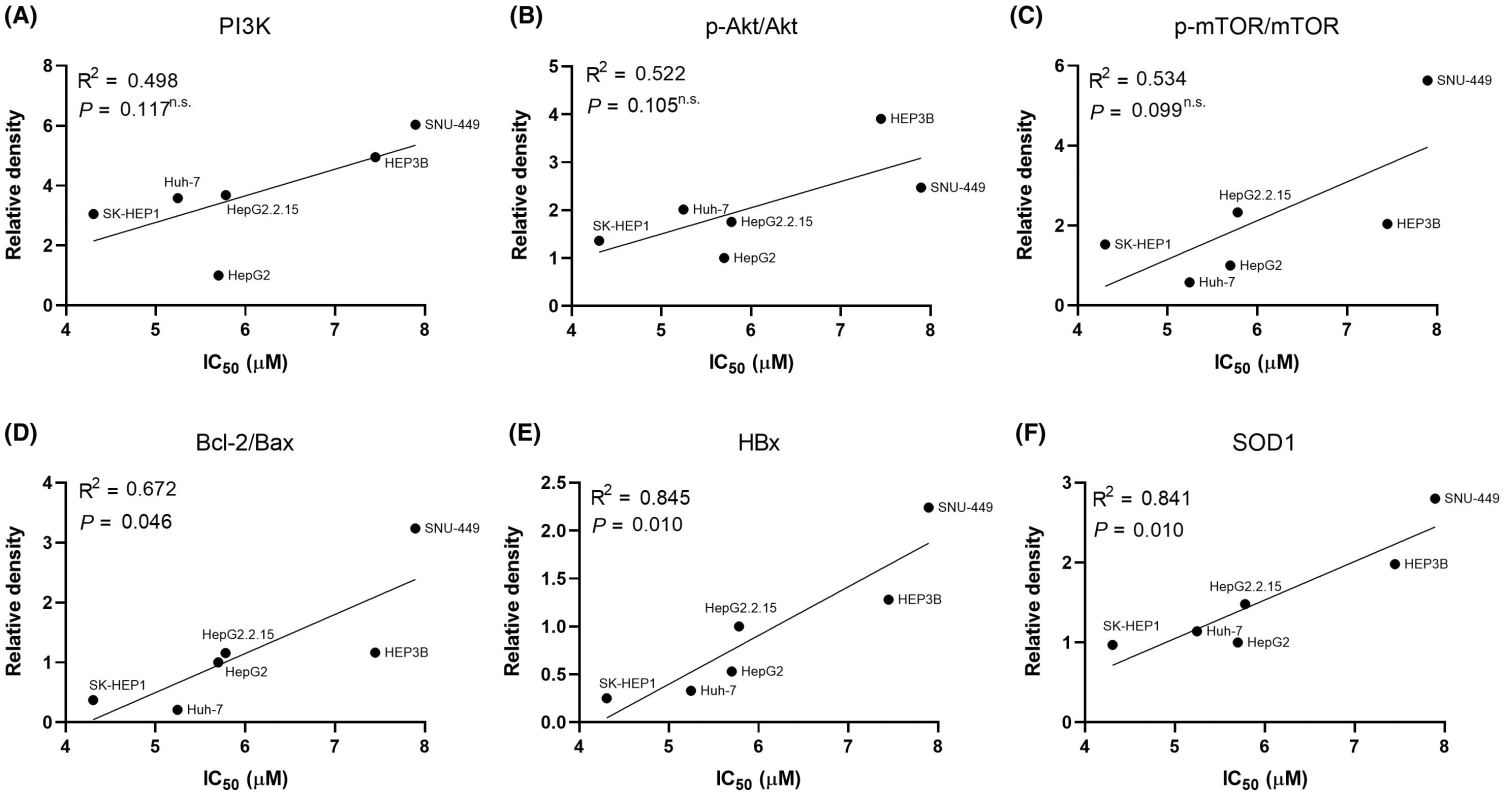
Correlation between IC_50_ of sorafenib and the activity of PI3K/Akt/mTOR pathway, apoptosis‐related markers, and protein expression of HBx and SOD1. (A–F) The correlation between IC_50_ (μM) of sorafenib and the protein levels of PI3K (A), pAkt/Akt (B), p‐mTOR/mTOR (C), Bcl‐2/Bax (D), HBx (E), and SOD1 (F) in both non‐HBV HCC cell lines (HepG2, SK‐HEP1, Huh‐7) and HBV‐related HCC cell lines (HepG2.2.15, HEP3B, and SNU‐449). A P‐value of less than 0.05 was considered statistically significant.

Additionally, HBV‐positive HCC cells showed elevated levels of key components in the PI3K/Akt/mTOR pathway including phosphorylated Akt (p‐Akt) and Akt ratio (Figure 2B,D). Similarly, the levels of phosphorylated mTOR (p‐mTOR) and the mTOR ratio were elevated in these cells (Figure 2E). Furthermore, the Bcl‐2/Bax ratio, an indicator of anti‐apoptotic status,^21^ was notably increased in HBV‐infected cells (Figure 2F). While statistical significance was not reached for all parameters, it is worth noting that the levels of PI3K were higher in HBV‐positive cells (4.89 ± 0.68) compared to HBV‐negative cells (2.54 ± 0.79) (*p =* 0.087). Similarly, the p‐Akt/Akt ratio was numerically higher in HBV‐positive cells (2.71 ± 0.63) compared to HBV‐negative cells (1.46 ± 0.30) (*p =* 0.148). The p‐mTOR/mTOR ratio was also numerically higher in HBV‐positive cells (2.71 ± 0.63) compared to HBV‐negative cells (1.46 ± 0.30) (*p =* 0.124). However, the Bcl‐2/Bax ratio was significantly higher in HBV‐positive cells (1.82 ± 0.71) compared to HBV‐negative cells (0.53 ± 0.24) (*p =* 0.042). While not all parameters reached statistical significance, it is noteworthy that all HBV‐containing cell lines exhibited indications of an activated PI3K/Akt/mTOR pathway and inhibited apoptosis‐related mechanisms. These factors are well‐established contributors to resistance against SF. Moreover, our data revealed positive correlations between PI3K/Akt/mTOR activation and anti‐apoptotic mechanisms with SF IC_50_ values in HCC cell lines (Figure 3A–D). Given the positive correlation between the IC_50_ of SF and the levels of HBx protein and SOD1 protein in HCC cell lines (Figure 3E,F), it can be assumed that cells with high HBx expression tend to exhibit elevated SOD1 levels and that both factors may be associated with SF resistance.

Collectively, our research indicates that in HCC cells with HBV, two things happen: the PI3K/Akt/mTOR pathway gets activated and the mechanisms related to cell death get inhibited. These changes make the cells more resistant to SF, a common treatment. So, having HBV seems to make HCC cells tougher to treat with SF by turning on the PI3K pathway and turning off cell death mechanisms.

### 3.3 SOD1 suppression enhances SF‐mediated cell death in HBV‐infected HCC cells

**FIGURE 4:**
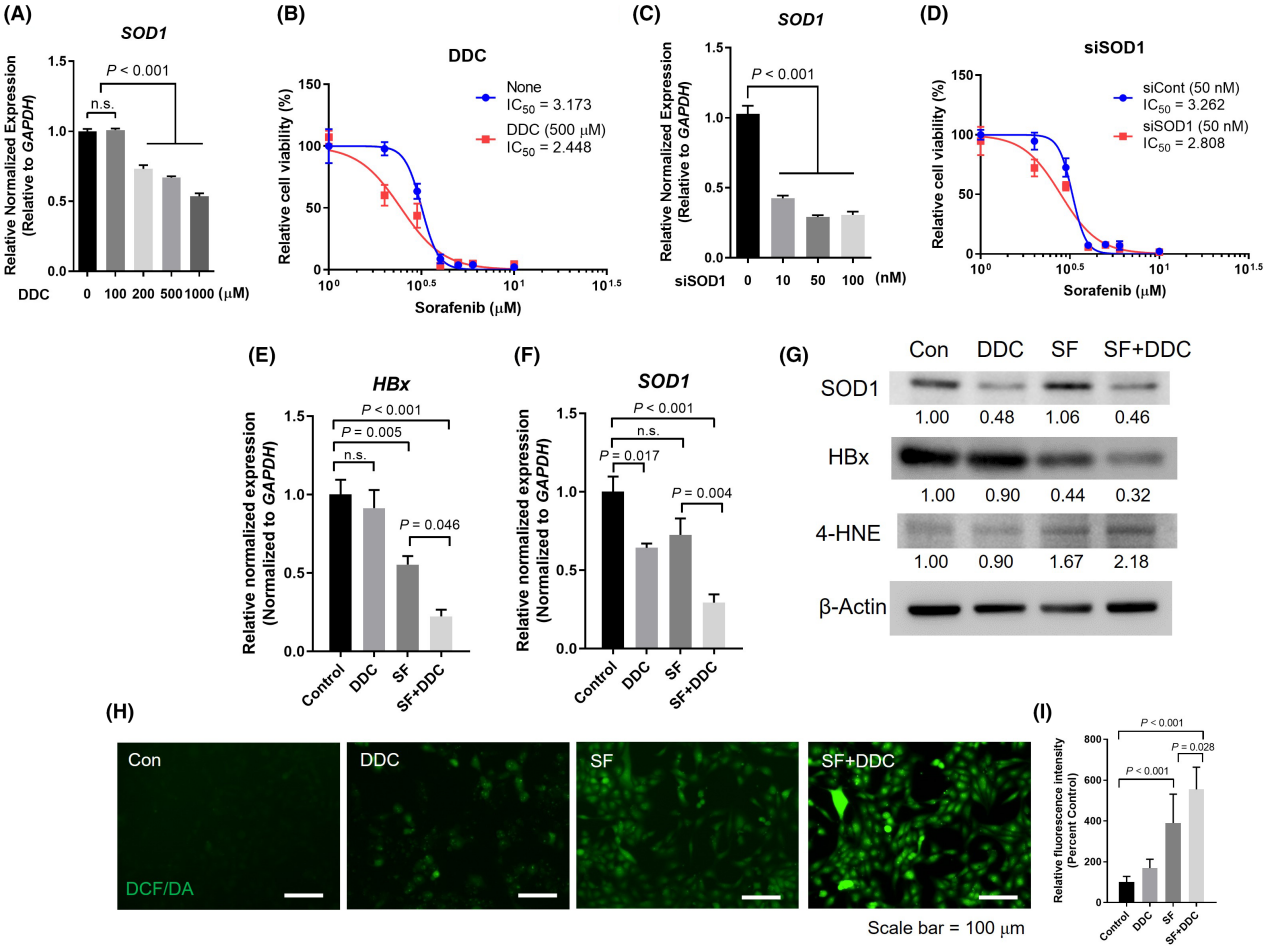
Examination of DDC's effects on HepG2.2.15 cells in the presence or absence of sorafenib Treatment. (A, B) SOD1 expression in HepG2.2.15 cells after 24 h treatment with DDC at various concentrations (A) and the corresponding assessment of relative cell viability (B). (C, D) SOD1 expression in HepG2.2.15 cells after 24 h treatment of siSOD1 (C) and the corresponding evaluation of relative cell viability (D). Significance was determined using one‐way ANOVA with Bonferroni's multiple comparisons test, with *p* < 0.05 considered statistically significant. (E, F) Gene expression levels of HBx (E) and SOD1 (F) in HepG2.2.15 cells that were treated with DDC, either alone or in combination with sorafenib. (G) Western blot analysis of SOD1, HBx, and 4‐HNE in HepG2.2.15 cells that underwent the same treatments, with the relative protein densities presented. (H, I) Cellular ROS staining using the H_2_DCFDA assay in cells treated with DDC, with or without sorafenib. It includes both the staining results (H) and a quantification of the relative fluorescence intensity (I). The Significance was determined using one‐way ANOVA with multiple comparisons test, with *p* < 0.05 considered statistically significant.

Building upon our findings, we investigated the potential of DDC, a derivative of disulfiram known for its SOD inhibition properties, to reduce SOD1 expression in HepG2.2.15 cells. DDC treatment effectively suppressed SOD1 gene expression (Figure 4A). This intervention resulted in a significant decrease in the IC_50_ of SF (Figure 4B). Furthermore, to investigate the inhibition of SOD1 more comprehensively, we utilized siRNA‐based suppression, which significantly increased SF‐induced cell death in HepG2.2.15 cells (Figure 4C,D).

In summary, our results highlight the potential of targeting SOD1 expression, either through DDC or siRNA‐based suppression, to significantly enhance the effectiveness of SF in inducing cancer cell death. This suggests a promising approach for adjunct therapy to enhance the effectiveness of SF treatment.

### 3.4 Synergistic effects of SF and DDC on ROS accumulation

Based on our previous findings, which showed increased levels of SOD1 in HBV‐positive HCC cell lines and the inhibitory effect of DDC on SOD1 expression, we aimed to examine whether the addition of DDC could enhance the effectiveness of SF in inducing cell death in HCC. In our combined treatment approach, we observed a significant reduction in both HBx and SOD1 expression at both the mRNA and protein levels (Figure 4E, F, and G). Furthermore, we observed an increase in the expression of the lipid ROS marker, 4‐hydroxynonenal (4‐HNE), in the group receiving the combined treatment (Figure 4G). Our analysis using H_2_DCFDA further revealed the highest ROS expression in the group treated with DDC in combination with SF (Figure 4H). Although the area of FITC‐positive cells showed an increase in all three experimental groups–DDC, SF and SF + DDC, it is noteworthy that the SF + DDC group displayed the highest fluorescent intensity (Figure 4I). These findings strongly support our hypothesis that the combination of SF and DDC synergistically inhibits HBx expression and leads to an accumulation of cellular ROS levels. Consistent with our previous data, which clearly demonstrated a strong correlation between cellular SOD1 expression and cellular HBx levels, the reduction in SOD1 may be intricately linked to HBx expression. However, it should be noted that while the inhibition of HBx can be associated with reduced SOD1 levels, the inhibition of SOD1 does not necessarily lead to the inhibition of HBx, as demonstrated in the case of SF‐alone treatment.

### 3.5 Combination of DDC and SF increases cell death without augmenting apoptosis

**FIGURE 5:**
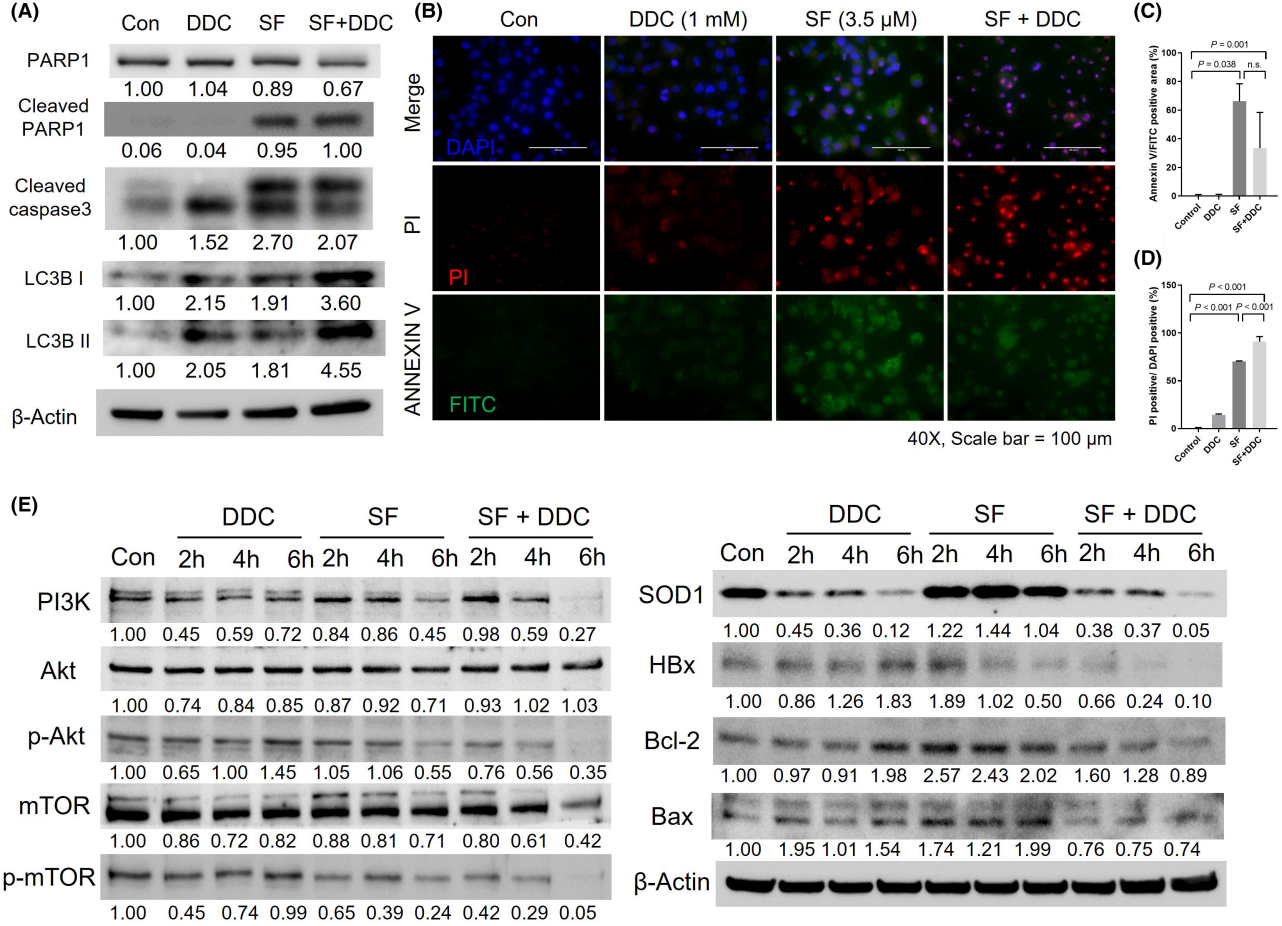
Examination of cell death in HepG2.2.15 cells subjected to DDC treatment with or without sorafenib. (A) Western blot analysis of apoptosis‐related proteins (PARP1, cleaved PARP1, cleaved caspase3) and an autophagy‐related protein (LC3B) in HepG2.2.15 cells that were treated with DDC, either alone or in combination with sorafenib. (B) Fluorescence imaging analysis for apoptosis using Annexin V‐FITC/PI staining in HepG2.2.15 cells that underwent the same treatments. (C, D) A quantification of the Annexin V‐FITC positive area (%) (C), and the ratio of PI‐positive cells to DAPI‐positive cells (D). (E) Evaluation of the PI3K/Akt/mTOR pathway and Bcl‐2/Bax levels in cells treated with DDC, SF, or SF + DDC at various time points (up to 6 h). The significance of the results was determined using one‐way ANOVA with Bonferroni's multiple comparisons test, with a *p*‐value of less than 0.05 considered statistically significant.

**FIGURE 6:**
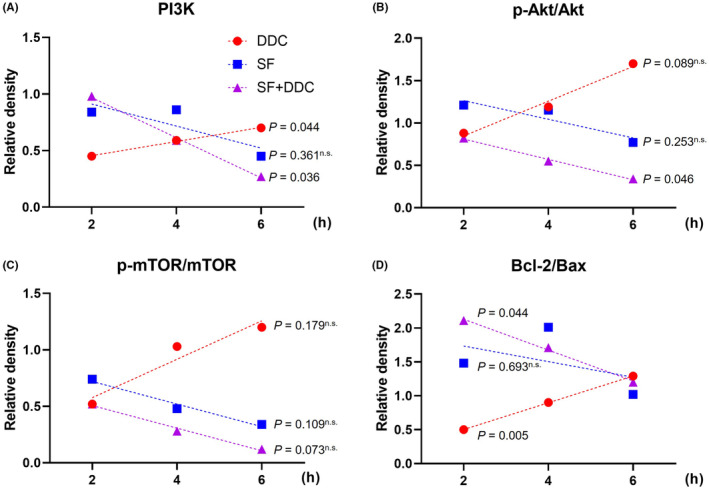
Temporal alterations in PI3K/Akt/mTOR pathway and Bcl‐2/Bax levels in DDC, SF, and SF + DDC groups. (A–D) The time‐dependent changes in the levels of PI3K (A), pAkt/Akt (B), p‐mTOR/mTOR (C), and Bcl‐2/Bax (D) in HepG2.2.15 cells. The cells were treated with DDC, sorafenib (SF), or the combination (SF + DDC) at different time points (2 h, 4 h, and 6 h). A *P* value less than 0.05 was considered statistically significant.

We found a significant increase in cell death in HepG2.2.15 cells when treated with the combination of SF and DDC. This prompted us to conduct further analysis to determine whether the increased cell death could be attributed to the ability of SF to induce apoptosis in cancer cells. Interestingly, we did not observe an increase in cleaved PARP1 or cleaved Caspase‐3, which are typical markers of apoptotic pathway activation (Figure 5A). Likewise, the autophagy marker LC3B remained unchanged in response to the combination therapy (Figure 5A). Upon further examination using Annexin V and PI fluorescence imaging, we observed an increase in cell death without a concurrent rise in cellular apoptosis (Figure 5B). Annexin V‐FITC‐positive cells were most abundant under SF‐alone conditions (Figure 5C), while PI‐positive cells peaked in the SF + DDC combination treatment (Figure 5D). These results collectively indicate that the reduction in cell viability observed with SF + DDC treatment is not primarily due to apoptotic cell death. To gain insights into the underlying mechanisms, we conducted a comprehensive analysis of the activation of the PI3K/Akt/mTOR pathway (Figure 5E). Our results demonstrated that the combination treatment of SF and DDC effectively inhibited the activation of this pathway. Notably, DDC treatment alone effectively suppressed SOD1 protein expression, while SF alone had a slight stimulatory effect on SOD1 expression. Additionally, the presence of the HBx protein was strongly suppressed by the combined treatment (Figure 5E). Markers associated with PI3K/Akt/mTOR pathway activation, including PI3K, the p‐Akt/Akt ratio, and the p‐mTOR/mTOR ratio, displayed a consistent upward trend over the course of drug treatment (up to 6 h), with the combined therapy exhibiting the most pronounced effects (Figure 6A–C). We also observed inhibition of both Bcl‐2, an anti‐apoptotic protein, and Bax, a pro‐apoptotic protein, as a result of the combination therapy of DDC and SF. Bcl‐2 levels showed a slight increase when treated with SF alone but were decreased by SF + DDC. Bax protein also exhibited a slight increase in the SF alone group and a decrease in SF + DDC conditions. Nevertheless, the Bcl‐2/Bax ratio, which indicates an anti‐apoptotic response, consistently decreased in response to the SF + DDC combined therapy (Figure 6D).

In conclusion, our findings suggest that the combination therapy of SF and DDC enhances cell death in HepG2.2.15 cells through multiple mechanisms. This combination treatment effectively inhibits the expression of SOD1, which in turn affects the accumulation of intracellular ROS and inhibits the anti‐apoptotic response of HBV‐infected cells during SF treatment. Moreover, it exerts significant effects on HBx protein levels and the activation of the PI3K/Akt/mTOR pathway, ultimately contributing to increased cell death.

## 4 COMBINING SF AND DDC LEADS TO TUMOUR VOLUME REGRESSION IN ORTHOTOPIC HCC XENOGRAFT MICE

**FIGURE 7:**
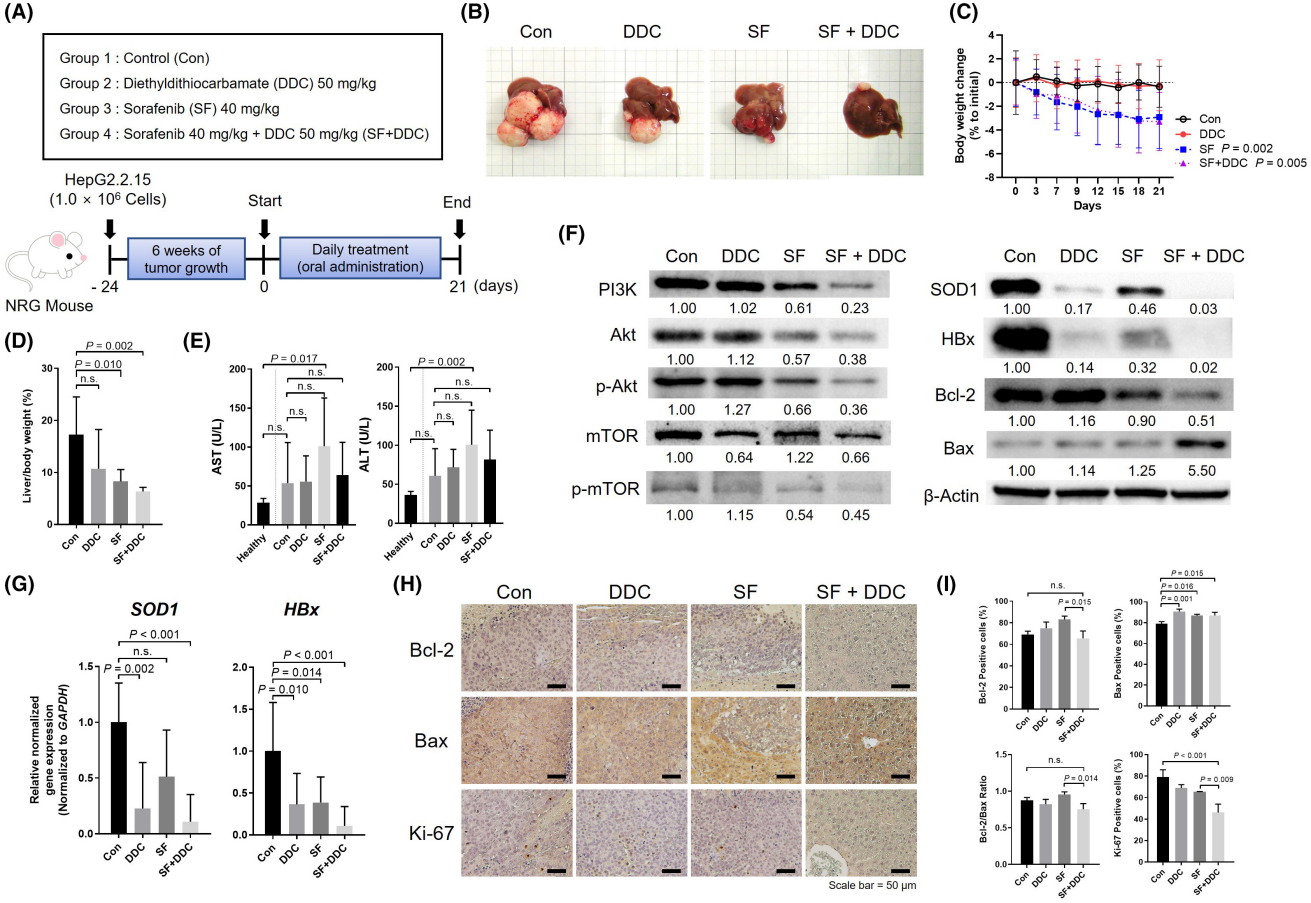
Analysis of tumour xenograft mice subjected to DDC treatment with or without sorafenib. (A) Schematic diagram of the experimental design. (B) Representative images of mice from each group: HCC control, Sorafenib only, DDC only, and combined Sorafenib + DDC (SF + DDC). (C) Graph showing the percentage of body weight change relative to the initial weight for each group. (D) Liver‐to‐body weight ratio (%) for each group. (E) Levels of AST and ALT enzymes in each group, indicating liver function. (F) Western blot images displaying the relative protein densities of PI3K, Akt, mTOR, Bcl‐2, Bax, SOD1, and HBx in liver tumours from each group. (G) Gene expression levels of SOD1 and HBx in tumours from each group. (H) Representative immunohistochemistry (IHC) images showing Bcl‐2, Bax, and Ki‐67 staining in liver sections of xenografted mice. (I) Quantification of positive staining areas for Bcl‐2, Bax, Ki‐67, and the Bcl‐2/Bax ratio in the liver sections. Statistical analysis was performed using one‐way ANOVA with Bonferroni's multiple comparisons test. A *p*‐value of less than 0.05 was considered statistically significant.

To evaluate the anticancer efficacy of combining DDC with SF, we established an orthotopic HCC xenograft model using NRG mice and the HepG2.2.15 cell line. After allowing the implanted tumour cells to grow for 6 weeks, we randomly divided the mice into four groups, each consisting of eight mice. The groups were treated daily with either SF (40 mg/kg), DDC (50 mg/kg), a combination of both (SF + DDC), or no treatment. Tumour samples were harvested on the 21st day of treatment (Figure 7A). The results demonstrated that the combined treatment of SF and DDC (SF + DDC) led to the most significant regression in tumour volume compared to the other treatment groups (Figure 7B). Mice treated with SF alone or in combination with DDC (SF + DDC) experienced a significant decrease in body weight relative to their initial weight, primarily due to reduced tumour size (Figure 7C). Notably, the percentage of liver weight relative to the total body weight was lowest in the combined treatment group (Figure 7D). The levels of AST and ALT enzymes did not significantly increase in the groups treated with DDC or the SF + DDC therapy, indicating that these treatments did not exacerbate liver damage (Figure 7E). In the histological analysis to assess liver damage induced by the drugs, no significant increase in necrosis or inflammation in the mouse liver was observed (Figure S1). Western blot analysis revealed that the combination treatment significantly reduced the levels of SOD1, HBx, PI3K, p‐Akt and p‐mTOR, as well as Bcl‐2, which aligns with previous in vitro results. Remarkably, this combination treatment led to an increase in Bax protein, indicating enhanced apoptosis in the xenografted HBx expressing HCC (Figure 7F). The in vivo xenograft animal model also revealed that DDC treatment decreases gene expressions of SOD1 and HBx in HCC. Moreover, the combined treatment of DDC and SF effectively inhibits SOD1 as well as HBx (Figure 7G).

In the IHC analysis of liver sections from our orthotopic HCC xenograft mouse model, distinct expression patterns of Bcl‐2, Bax and Ki‐67 proteins were observed. The combination treatment of DDC and SF (SF + DDC) notably reduced the expression of Bcl‐2, an anti‐apoptotic protein, suggesting a decrease in anti‐apoptotic signals within the tumour environment. Conversely, the expression of Bax, a pro‐apoptotic protein, was significantly increased, indicating a shift towards apoptotic pathways in the treated tumours. Additionally, the proliferation marker Ki‐67 showed reduced expression in the combination treatment group, further supporting the efficacy of the treatment in reducing tumour cell proliferation (Figure 7H,I). These findings highlight the pro‐apoptotic and anti‐proliferative effects of the combined DDC and SF treatment, enhancing our understanding of its therapeutic impact on HBV‐related HCC.

### 4.1 Discussion

In the context of the global health challenge posed by liver diseases associated with HBV infection, the management of HBV‐related HCC remains a significant clinical hurdle. HBV‐related HCC is characterized not only by its promotion of cancer cell proliferation but also by its resistance to apoptosis, complicating effective treatment strategies. In this study, we explored the therapeutic potential of combining DDC with the traditional antineoplastic agent SF to treat HBV‐positive HCC.

Recent studies underscore the pivotal role of the mTOR pathway in oncogenesis, suggesting its broad implications across various cancers.^22^, ^23^, ^24^ These investigations reveal enhanced mTOR signalling in tumour environments, promoting proliferation and survival, a finding consistent with the mechanistic pathways observed in HBV‐related HCC.^25^, ^26^ This convergence of evidence supports the hypothesis that HBV infection directly modulates mTOR signalling, suggesting a targeted approach for therapeutic intervention. Further research should thus focus on the mTOR pathway's specific roles and regulatory mechanisms in HBV‐related HCC to develop more effective treatments tailored to this complex interplay of viral infection and cancerous progression.

Our study builds on these findings by exploring the effects of DDC, a compound known for its anticancer properties. The prior studies demonstrated that DDC triggers ROS‐dependent Bax and cytochrome c translocations, which are potentially pro‐apoptotic. Also, DDC has reported to inhibit caspase activation, mitochondrial membrane potential (ΔΨm) loss, and cell death in a ROS‐independent manner.^27^ Our pro‐apoptotic role of DDC aligns with previous studies on the use of DDC, and we have experimentally confirmed that DDC exerts this mechanism through the regulation of SOD1. Furthermore, we examined the therapeutic potential of combining DDC with SF, hypothesizing that this combination could effectively target the mTOR pathway. Our results indicate that the combination therapy disrupts the PI3K/Akt/mTOR signalling pathway and promotes apoptosis by increasing ROS levels. This disruption leads to reduced tumour viability and enhanced sensitivity to SF, as evidenced by the synergistic suppression of tumour growth in xenograft models. Therefore, our study suggests that DDC, through its pro‐apoptotic signals and ability to inhibit mTOR pathway components, holds significant promise as a complementary agent to SF.

In alignment with our findings, recent studies have highlighted the therapeutic potential of combining metformin, a commonly prescribed diabetes medication that improves insulin sensitivity, with SF in treating HCC. Metformin enhances the efficacy of SF by modulating the ATF4/STAT3 pathway, leading to increased ROS and induction of ferroptosis, thereby reducing SF's IC50 value.^28^, ^29^ Additionally, the combination has been shown to increase apoptotic cell death in HepG2.2.15 cells through alterations in the mTOR pathway, suggesting a promising approach for improving HCC treatment outcomes.^30^ Given the outcomes of metformin‐SF combination therapies, the DDC‐SF combination could be another novel strategy to overcome resistance in HBV‐related HCC, potentially optimizing therapeutic outcomes. Moreover, considering the combined treatment of DDC and SF has increased cellular levels of lipid ROS, such as 4‐HNE, DDC‐SF combination therapy can modulate the inhibition of the diverse pathways including PI3K/Akt pathway, via modulating SOD1 and related cellular antioxidant mechanisms. Further investigation is needed to determine the specific type of cell death induced by DDC as well as the underlying mechanisms and pathways involved. Considering potential avenues for further exploration, it is worth noting that medications with inhibitory effects on SOD1 hold promise for elucidating similar effects.

The innovative potential and novelty of our work lie in the strategic inhibition of multiple pathways to combat the complex interplay of viral gene expression and cancer progression through antioxidant gene, SOD1. Overall, our study provides a compelling rationale for the continued development of combination therapies targeting key signalling pathways to overcome resistance mechanisms in HBV‐related HCC. This approach represents a novel therapeutic avenue by leveraging the synergistic effects of DDC and SF, particularly through the modulation of the PI3K/Akt/mTOR pathway. By addressing the specific vulnerabilities of HBV‐positive HCC cells, our research paves the way for more effective and targeted treatment strategies. Further investigations are warranted to elucidate the precise molecular mechanisms underlying this synergistic effect and to evaluate the potential clinical applications of this combination therapy.

## AUTHOR CONTRIBUTIONS

**Jooyoung Lee:** Conceptualization (lead); data curation (lead); formal analysis (lead); investigation (lead); methodology (lead); visualization (lead); writing – original draft (lead). **Jiye Kim:** Conceptualization (equal); data curation (supporting); investigation (equal); methodology (equal); validation (lead); visualization (supporting). **Ryunjin Lee:** Investigation (supporting). **Eunkyeong Lee:** Investigation (supporting). **Hye‐In An:** Investigation (supporting). **Yong‐Jae Kwon:** Investigation (supporting); resources (supporting). **Hana Jin:** Investigation (supporting); resources (supporting). **Chan‐Gi Pack:** Resources (supporting). **Inki Kim:** Resources (supporting). **Young‐In Yoon:** Resources (supporting); supervision (equal). **Gil‐Chun Park:** Resources (supporting); supervision (equal). **Eun‐Kyoung Jwa:** Resources (supporting). **Jae Hyun Kwon:** Funding acquisition (equal); resources (supporting). **Jung‐Man Namgoong:** Resources (supporting); supervision (equal). **Gi‐Won Song:** Resources (supporting); supervision (equal). **Shin Hwang:** Resources (supporting); supervision (equal). **Eunyoung Tak:** Conceptualization (equal); funding acquisition (equal); project administration (equal); supervision (equal); visualization (equal); writing – review and editing (equal). **Sung‐Gyu Lee:** Resources (supporting); supervision (equal).

## FUNDING INFORMATION

This work was supported by the National Research Foundation of Korea through grants 2021R1G1A1093955 awarded to JHK, and grants 2015K1A4A3046807, 2017R1D1A1B04032429, and 2022R1A2C2006141 awarded to ET, as well as the Asan Institute for Life Sciences, Asan Medical Center, Seoul, South Korea, through grant 2023IP0028.

## CONFLICT OF INTEREST STATEMENT

The authors have no conflicts of interest to declare.

## Supporting information


Figure S1.

**Table S1.**.

## Data Availability

The data that supports the findings of this study are available in the supplementary material of this article.
