# Supplementary material for: SOD1 inhibition enhances sorafenib efficacy in HBV‐related hepatocellular carcinoma by modulating PI3K/Akt/mTOR pathway and ROS‐mediated cell death
Source: J Cell Mol Med. 2024 Jul 21;28(14):e18533. doi: 10.1111/jcmm.18533 (PMC11260765; doi:10.1111/jcmm.18533)
Supplement: Supplementary file 1 — Figure S1. [file JCMM-28-e18533-s001.docx]

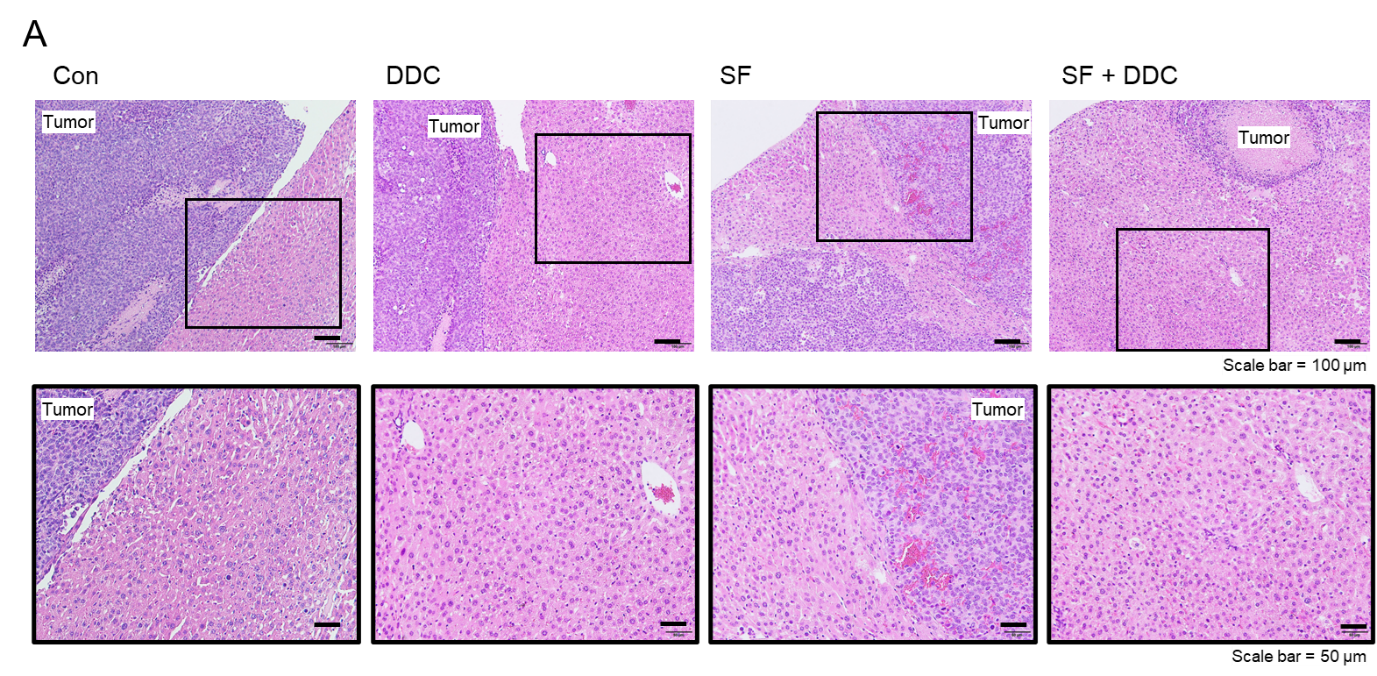


**Supplementary Figure 1. Histological analysis of liver in xenograft mouse models.**

Hematoxylin and Eosin (H&E) staining of liver sections from HBV-related HCC orthotopic xenograft mice across different treatment groups (Control, DDC, SF, and SF+DDC). The images are presented at 100x and 200x magnifications. The scale bars represent 100 µm and 50 µm, respectively.
